# A hydrogel platform delivering mesenchymal stem cell lysate for potential myocardial tissue repair

**DOI:** 10.3389/fbioe.2026.1855161

**Published:** 2026-07-31

**Authors:** Ziwei Du, Qi Zhang, Qiuhua Wang, Paul Santerre, Tianyi Ma, Peng Ma, Xiaoqing Zhang

**Affiliations:** 1 International Joint Laboratory of Biomaterials and Tissue Regeneration, School of Basic Medicine, Binzhou Medical University, Yantai, Shandong, China; 2 Institute of Biomedical Engineering, University of Toronto, Toronto, ON, Canada; 3 Li Ka Shing Faculty of Medicine, The University of Hong Kong, Pokfulam, Hong Kong SAR, China

**Keywords:** adipose-derived stem cells, cell-free therapy, hydrogel, lysates, myocardial infarction

## Abstract

**Introduction:**

Myocardial infarction is one of the most significant causes of death worldwide and the limited regenerative capacity of the myocardium often results in poor recovery after injury. Stem cell therapy has some promise for myocardial tissue repair, but clinical translation is hindered by poor cell survival and safety concerns, prompting the investigations of cell-free strategies such as stem cell-derived secretome or lysate.

**Methods:**

In this study, a quaternized chitosan/tannic acid hydrogel was fabricated and characterized for its crosslinking stability, injectability and degradation kinetics. In addition, mouse adipose-derived mesenchymal stem cells (mADSCs) were isolated, characterized, and processed into cell-free lysate. The mADSCs lysate was then analyzed by TEM, NTA, Western blot and enzyme-linked immunosorbent assay.

**Results:**

It was found that the mADSCs lysate contained abundant vesicles and a broad panel of cytokines/growth factors with anti-inflammatory, pro-angiogenic and antioxidant properties. Encapsulating the lysate within the hydrogel showed sustained release. The hydrogel-based delivery of mADSCs lysate could promote high metabolic activity and good cell number increases of H9c2-cardiomyocytes, L929-fibroblasts and human umbilical vein endothelial cells, demonstrating great potential for myocardial tissue repair applications.

**Discussion:**

By integrating the regenerative potential of ADSCs lysate with this biocompatible, injectable hydrogel platform, this combined strategy circumvents the potential safety issues associated with live cell transplantation and offers a scalable, potential cell-free delivery strategy for myocardial repair-related applications, laying a foundation for future *in vivo* studies and further translational evaluation.

## 1 Introduction

Myocardial infarction (MI) remains one of the leading causes of death across the globe and a major contributor to significantly reduced life quality and productivity (Krittanawong et al., 2023). Myocardial tissue has limited self-regeneration capacity, causing poor myocardium recovery post-MI (Zhu et al., 2025). Stem cell therapy has gained great popularity for myocardial tissue repair and regeneration, as it could have the potential to stimulate tissue angiogenesis, enhance perfusion and promote new myocardial tissue formation (Carbone et al., 2021). Several stem cell types, for instance embryonic stem cells (ESCs), induced pluripotent stem cells (iPSCs) and mesenchymal stem cells (MSCs), have attracted attention for myocardial tissue repair (Carbone et al., 2021). Among those, MSCs have become a widely explored cell source for treating MI and their therapeutic effects have been identified to be primarily attributed to paracrine signaling (Sid-Otmane et al., 2020; Rayat Pisheh and Sani, 2025). However, the harsh microenvironment at the tissue damage site often leads to poor survival of transplanted MSCs (Yan W. et al., 2024). To overcome the limitations associated with live MSCs transplantation, cell-free approaches utilizing MSCs-derived secretome or MSCs lysate have emerged as promising alternatives.

MSCs lysate is defined as a liquid that contains the intracellular microvesicles and growth factors/cytokines of lysed MSCs. Acting as endogenous molecular factories, MSCs often harbor a richer repertoire of anti-apoptotic, anti-inflammatory and pro-angiogenic growth factors/cytokines compared to MSCs secretome alone (Zununi Vahed et al., 2025). In the past 5 years, MSCs lysate has been investigated for its repair and regenerative potential across diverse tissue types, including enhancing *in vitro* embryonic development (Manabe et al., 2024), alleviating skin photoaging (Duan et al., 2023), repairing cavernous nerve injury (Mori et al., 2022), ameliorating inflammatory bowel disease (Nishikawa et al., 2021) and promoting liver regeneration (Herencia et al., 2015; Satilmis et al., 2022). Although no studies to date have investigated the use of MSCs lysate-derived vesicles for repairing myocardial injury, the aforementioned multiple studies suggest that MSCs lysate-derived vesicles also hold considerable therapeutic potential for myocardial tissue repair and regeneration.

Adipose-derived MSCs (ADSCs) stand out among the different MSC subtypes (i.e., bone marrow-, hair follicle-, umbilical cord blood-, dental pulp-derived, etc.) for treating cardiovascular diseases, due to their ease of isolation, significant abundancy, great expandability *in vitro*, good differentiation potential and robust paracrine signaling capabilities (Qin et al., 2023; Yang et al., 2024). Previous investigations have revealed that ADSC-derived exosomes alleviated myocardial ischemia-reperfusion injury (Lee et al., 2025; Xu et al., 2025), and that ADSC secretions exerted cardioprotective effects partially via microRNA-221 (Lee et al., 2021). Despite these advances, whether ADSC lysates have any therapeutic potential for post-MI myocardial tissue repair and regeneration remains unexplored.

The bioactive components in MSCs lysate mainly include microvesicles, cytokines, growth factors and microRNAs, which often suffer from poor stability in the *in vivo* microenvironment, limiting its therapeutic efficacy (Luo et al., 2022). Accordingly, biocompatible and biodegradable delivery platforms are critical for effective *in vivo* application of MSCs lysate. Hydrogels have gained prominence in myocardial tissue repair and regeneration due to their similarity to native extracellular matrix and water-retention capability (Xu et al., 2024). At present, hydrogel-based therapies for MI have gradually advanced from preclinical animal studies towards clinical translation. However, only a limited number of biomaterials have entered formal clinical trials. Among them, alginate hydrogel-based systems represent the most mature candidates (Frey et al., 2014; Ruvinov and Cohen, 2016), yet they suffer from drawbacks including batch-to-batch raw material variations, slow *in vivo* degradation, risk of local calcium overload, lack of intrinsic bioactivity and unsatisfactory growth factor delivery performance (Liberski et al., 2016). Collagen hydrogels can closely mimic native extracellular matrix and exhibit excellent cell adhesion capacity, but their mechanical properties are relatively weak. Polyethylene glycol (PEG)-based hydrogels possess widely tunable mechanical properties, yet they are highly bioinert (Patel and Patel, 2024). In comparison, the QCS-TA hydrogel delivery system not only favorably mimics the extracellular matrix structure, enables long-term sustained release of cytokines, but also inherently integrates multiple bioactive functions such as anti-inflammation and antioxidation (Yan C. et al., 2024), endowing it with broader prospects for clinical translation. A previous work showed that acellular chitosan-based hydrogels alone can exert beneficial effects on myocardial repair (Shu et al., 2015) and their highly porous structure could enable sustained drug release (Xu et al., 2024). Recent evidence also suggested that chitosan-based hydrogels can serve as effective vehicles for carrying exosomes to promote cardiac tissue repair (Yan C. et al., 2024). However, the utilization of chitosan-based hydrogels loaded with MSCs lysate for potential myocardial tissue repair and regeneration remains uninvestigated.

This study aimed to investigate the feasibility of delivering ADSCs lysate using an injectable quaternized chitosan/tannic acid (QCS/TA) hydrogel for myocardial tissue repair. The QCS/TA hydrogel was fabricated via simple blending as previously established (Guo et al., 2022b; 2022a), and then it was characterized by Fourier-transform infrared spectroscopy, rheological analysis, injectability testing, and *in vitro* degradation assays. Additionally, mouse ADSCs (mADSCs) were isolated from the inguinal subcutaneous adipose tissue and characterized for their colony forming ability, immunophenotype and tri-lineage differentiation capability. ADSCs lysate was prepared using hypotonic treatment combined with repeated freeze-thaw cycles. Afterwards, the ADSCs lysate was characterized for its derived vesicle content and cytokines/growth factors profile by TEM, NTA, immunofluorescence staining, Western blot and enzyme-linked immunosorbent assay. Moreover, the ADSCs lysate-derived vesicles were encapsulated into the QCS/TA hydrogel to examine its *in vitro* release profile. Finally, in order to comprehensively assess the potential of this QCS/TA-based ADSCs lysate delivery platform for myocardial tissue repair, the effects of QCS/TA, ADSCs lysate, as well as the combination of the two on cell number (DNA assay) and cell metabolic activity (WST-1 assay) of H9c2, L929 and HUVECs were investigated. Those 3 cell types were particularly selected to represent key aspects of the post-MI myocardial tissue microenvironment: H9c2 cardiomyocytes serving as the primary functional cells of the myocardium, L929 fibroblasts that can be essential to the fibrotic responses following MI and HUVECs that play an important role in tissue angiogenesis post-MI. Evaluating the effects of the QCS/TA-based ADSCs lysate delivery platform on the proliferation and cell metabolic activities of cardiomyocytes, fibroblasts and endothelial cells could provide significant insights into cardiomyocyte viability, fibrotic modulation and angiogenic potential within the myocardium, which are all key processes governing cardiac repair (Foglio et al., 2024).

## 2 Materials and methods

All materials were purchased from Sigma Aldrich unless stated otherwise.

### 2.1 Preparation of QCS/TA hydrogel

To prepare the QCS/TA hydrogel, 3% QCS (w/v) (Yuanye) and 5% TA (w/v) (Aladdin) solutions were prepared separately by dissolving each powder in deionized water and stirred thoroughly. Then, 1:1 QCS: TA solutions were prepared, yielding a hydrogel with a final concentration of 1.5% (w/v) QCS and 2.5% (w/v) TA, as established by previous studies (Guo et al., 2022b; 2022a). This 1:1 QCS/TA volume ratio was selected based on the prior formulation because it provided rapid gelation, injectable handling and stable QCS/TA crosslinking without requiring additional chemical crosslinkers.

### 2.2 Fourier-transform infrared (FT-IR)

QCS powder (400 mg), TA powder (400 mg) and the powder obtained by freeze-drying and grinding 400 μL of the QCS/TA hydrogel were prepared for FT-IR spectroscopy (Frontier Mid-IR FTIR0, Perkin-Elmer). FT-IR spectra were recorded (wavenumber range: 400–4,000 cm^-1^).

### 2.3 Dynamic strain sweep rheology

The rheological property of the hydrogel was characterized using a rheometer (AR-G2, TA Instruments) equipped with a 40 mm-D cone-plate geometry (1° cone angle). A strain-sweep test was performed (37 °C) with a fixed angular frequency (10 rad s^-1^). During the experiment, the applied strain increased from 0.1% to 1000% to determine the strain failure point of the hydrogel samples.

### 2.4 Gelation time and injectability test

Hydrogel gelation time was determined using the vial inversion method. Specifically, QCS solution (1 mL) and TA solution (1 mL) were thoroughly mixed in a transparent glass vial and incubated (37 °C). Successful gelation was defined as the absence of observable fluid flow 30 s after inverting the vial.

To assess injectability, 600 μL hydrogel was transferred to a 10 mL syringe and extruded through a 25G needle onto a flat surface to confirm injectability and writability.

### 2.5 *In vitro* degradation assessment of the hydrogel

The hydrogel was immersed in PBS (pH 7.4) at 37 °C for 0, 1, 3, 7, 14, 21, and 28 days. For all time points, the hydrogel was frozen at −80 °C for 12 h, completely lyophilized with a vacuum freeze-dryer, and the dry weights of the samples were measured.

SEM was used to observe the internal micro-structures of the hydrogel. The freeze-dried hydrogel samples were immersed into liquid nitrogen and fractured to obtain the cross-sections, which were sputter-coated with gold and then observed with SEM (EVO LS153040702, Zeiss).

### 2.6 Isolation and culture of mADSCs

The study was conducted in accordance with the Regulations of Binzhou Medical University and approved by the Ethical Review Committee of Binzhou Medical University (Approval No. 2024–248, June 12, 2024). KM mice used in this study were obtained from Jinan Pengyue Biotechnology Co., Ltd. The animals were housed under pathogen-free conditions with free access to food and water and the animal lab temperature was maintained at 24 °C ± 2 °C. Primary mADSCs were isolated from the inguinal subcutaneous adipose tissue of 8-week old mice. Specifically, an abdominal incision was made in the mice and the inguinal fat pads were carefully dissected with forceps and tissue scissors, and then immediately placed in pre-cooled (4 °C) PBS. The adipose tissue was transferred to a 15 mL centrifuge tube, rinsed three times with PBS containing 1% penicillin-streptomycin, and then minced into a paste utilizing ophthalmic scissors. The thoroughly minced tissue was then digested with 2–3 volumes of 0.1% type I collagenase (Biosharp) at 37 °C with shaking (120 rpm, 1 h). Tissue digestion was stopped by adding an equal volume of complete DMEM/F12 medium and the digested mixture was centrifuged to pellet the cells (1,500 rpm, 10 min). After discarding the supernatant, the cell pellet was resuspended in 5 mL DMEM/F12, filtered through a 70 μm cell strainer and collected into a 15 mL centrifuge tube. The cell filtrate was seeded into a T25 cell culture flask (Falcon). Those cells were labelled as passage 0 (P0) mADSCs.

### 2.7 Preparation of mADSCs lysate

mADSCs were maintained in DMEM/F12 medium supplemented with 10% FBS (37 °C, 5% CO_2_). Upon reaching 90% confluency, mADSCs were harvested using trypsin-EDTA solution (Solarbio) and washed three times with PBS to remove residual trypsin and medium. For mADSCs lysate preparation, 1 × 10^7^ mADSCs were resuspended in 1 mL deionized water and incubated for 30 min to induce osmotic lysis, following three freezing-thawing cycles at −80 °C to further disrupt the cellular contents. The lysate was then centrifuged (4,000 g, 30 min, 4 °C) and the final lysate solution was aliquoted and stored at −80 °C. All lysate samples were utilized for subsequent experiments within 1 month, avoiding repeated freeze-thaw cycles to minimize protein degradation and ensure experimental reproducibility.

### 2.8 Assessment of mADSCs lysate release by hydrogel

The mADSCs lysate was encapsulated in the QCS/TA hydrogel, which was then immersed in pH 7.4 PBS at 37 °C for 0 h, 6 h, 12 h, 24 h, 48 h, 96 h, and 168 h. The supernatant was collected at those different time points and the protein concentration of every single collected sample was measured utilizing a BCA protein assay kit (Bioswamp), according to the manufacturer’s specified instructions.

### 2.9 Colony-forming unit-fibroblast (CFU-F) assay

mADSCs were seeded in a 6-well plate at a density of 40 cells/well and cultured for 14 days in DMEM/F12 complete medium (supplemented with 16.5% FBS, 1% pen/strep and 2 mM L-glutamine). After culture, the mADSCs were fixed with 4% paraformaldehyde (20 min) and then stained with 3% crystal violet solution (10 min), and the representative colony images were captured using an inverted microscope.

### 2.10 Flow cytometry

mADSCs (P5) were harvested using trypsin-EDTA upon the cells reaching 90% confluency. The cells were washed three times using PBS to remove any residual culture medium or trypsin-EDTA. Fluorescent antibodies including FITC-CD29 (BioLegend), FITC-CD44 (BioLegend), PE-CD34 (BioLegend) and FITC-CD45 (BioLegend), were used to stain the mADSCs and the immunophenotype of mADSCs was analyzed by a flow cytometer (BD Canto II, BD Biosciences).

### 2.11 Trilineage differentiation

mADSCs were seeded in 96-well plates and induced to undergo adipogenic, osteogenic and chondrogenic differentiation upon reaching 80% confluency, as previously established by the authors’ group (An et al., 2025).

For adipogenic differentiation, mADSCs were cultured in adipogenic differentiation medium (10% FBS, 1% pen/strep, 1% L-glutamine, 0.2% insulin, 0.2% IBMX, 0.1% rosiglitazone, 0.1% dexamethasone), with a medium change every 2 days. After inducing for 21 days, differentiated mADSCs were stained with Oil Red O and examined under a microscope.

For osteogenic differentiation, mADSCs were cultured in osteogenic differentiation medium (10% FBS, 1% pen/strep, 1% L-glutamine, 0.2% ascorbic acid, 1% sodium β-glycerophosphate, 0.01% dexamethasone), with a medium change every 2 days. After inducing for 21 days, differentiated mADSCs were stained with Alizarin Red and examined under a microscope.

For chondrogenic differentiation, mADSCs were cultured in chondrogenic differentiation medium (10% FBS, 1% pen/strep, 0.1% proline, 0.3% ascorbic acid, 1% sodium pyruvate, 1% TGF-β1, 1% ITS), with a medium change every 2 days. After inducing for 21 days, differentiated mADSCs were stained with Alcian blue and examined under a microscope.

### 2.12 Transmission electron microscopy (TEM) and nanoparticle tracking analysis (NTA)

For TEM examination of the mADSCs lysate, the lysate samples were placed on carbon-coated grids and allowed to dry for 10 min (RT). The grids were then washed with PBS and stained with 4% (w/v) uranyl acetate for 2 min. The microstructure of the lysate was observed under TEM (Sense BSD, Zeiss) after drying. The particle size and concentration of the mADSCs lysate were determined by NTA (ZetaView S/N 23–950, Particle Metrix).

### 2.13 PKH26 staining

PKH26 staining was performed to evaluate the uptake of mADSCs lysate-derived vesicles by H9c2 cells. Vesicles were first isolated from the mADSCs lysate by ultracentrifugation (100,000 g, 70 min), and the supernatant of lysate was retained at 4 °C. The isolated vesicles were stained with PKH26 (Beyotime) and then washed twice by sequential ultracentrifugation and resuspension in PBS to thoroughly remove excess dye. The vesicle solution was reconstituted to 1 mg/mL, and then added to H9c2 cells (which were cultured in complete medium supplemented with 10% exosome-depleted FBS (Porcell) for 24 h) that were grown on tissue-culture glass coverslips (Neuvitro GG18PLL). The cells were then fixed with 4% paraformaldehyde at 4 °C (10 min), stained with DAPI (Solarbio) and examined under a fluorescence microscope (AxioScope 5, Zeiss Group). All staining procedures were performed in the dark.

### 2.14 Western blot (WB)

WB was performed to characterize cytokines/growth factors in the mADSCs lysate. The lysate was mixed with 1 mM phenylmethanesulfonyl fluoride (PMSF, Meilun) and protein concentrations were determined with BCA Protein Assay Kits (Bioswamp) according to the manufacturer’s protocol. For all samples, 40 μg total protein was loaded into each lane of 10%–15% PAGE gels to run gel electrophoresis. After the proteins were transferred from gels to the PVDF membranes, the membranes were washed twice with TBST buffer (24.2 g Trizma Base, 35.04 g NaCl, 4 mL Tween-20 for 4 L buffer volume, pH 7.5) and blocked with 5% skim milk (Beyotime) for 1 h to prevent nonspecific binding (at RT). The blots were then incubated with primary antibodies at 4 °C for overnight. The primary antibodies used include the following: HIF-1α (Immunoway, 1:1000), MMP9 (Proteintech, 1:1000), Nrf2 (Proteintech, 1:1000), VEGFA (Proteintech, 1:1000), PDGF-BB (Santacruz, 1:1000), TSG-6 (Servicebio, 1:800), IL-10 (Immunoway, 1:1000), FGF-2 (Santacruz, 1:1000) and SDF-1α (Epizyme, 1:1000). After incubation with HRP-conjugated secondary antibodies (1:2000 goat anti-mouse or goat anti-rabbit IgG in 5% milk), the protein signals were visualized with enhanced chemiluminescence reagent and imaged with a Tanon 5200 Multi imager.

### 2.15 Enzyme-linked immunosorbent assay (ELISA)

The concentrations of HIF-1α, MMP9, Nrf2, VEGFA, PDGF-BB, TSG-6, IL-10, FGF-2, and SDF-1α in the mADSCs lysate were determined using Bioswamp ELISA kits, according to the manufacturer’s protocols.

### 2.16 DNA quantification assay and WST-1 assay

At 24 h and 48 h time points, cells (H9c2, L929, and HUVECs) were lysed in 50 μL 0.05% Triton-EDTA lysis buffer (Solarbio) for 1 h, on ice. Samples were then vortexed for 30 s and incubated for 30 min at 65 °C, the process was repeated twice to ensure complete lysis, following 15,000 rpm centrifugation for 15 min (4 °C). DNA contents of the different samples were assessed by staining with 1 mg/mL Hoechst 33,258 dye (Solarbio). Fluorescence values were detected with a SpectraMax M2 plate reader (Molecular Devices) at 360 nm excitation and 460 nm emission wavelengths. DNA concentrations of the different samples were calculated against calf thymus DNA standards.

At 24 h and 48 h time points, cells (H9c2, L929, and HUVECs) were incubated with a 1:10 mixture of WST-1 reagent (Beyotime) and DMEM/F12 complete medium (37 °C, 1 h). Absorbance was measured at 450 nm using a plate reader (SpectraMax M2, Molecular Devices).

### 2.17 Data analysis

All data were analyzed with SPSS 22.0 software (SPSS Inc., Chicago, IL). Two groups’ comparisons were performed using an independent samples’ t-test, while multi-group comparisons were evaluated by ANOVA followed by Tukey’s or Dunnett T3 post-hoc test, where appropriate. All experiments were performed for at least three times and all data of the study are presented as mean ± SD, with statistical significance reported for a p-value < 0.05. More specifically, unless stated otherwise, all experiments were performed with at least three independent biological replicates and three technical replicates. Lysates were prepared from multiple independent animals and all experiments were repeated using independent cell preparations. Technical replicates refer to repeated measurements of the same biological sample. Normality was tested before ANOVA. Data following a normal distribution were analyzed using one-way ANOVA followed by post-hoc tests, while non-normally distributed data were analyzed using non-parametric tests, including the Mann-Whitney U test for two-group comparisons and the Kruskal-Wallis test followed by Dunn-Bonferroni post-hoc comparisons for multiple groups. Exact sample sizes (n values) for each figure are provided in the respective figure legends.

## 3 Results

### 3.1 Characterization of the QCS/TA hydrogel

**FIGURE 1:**
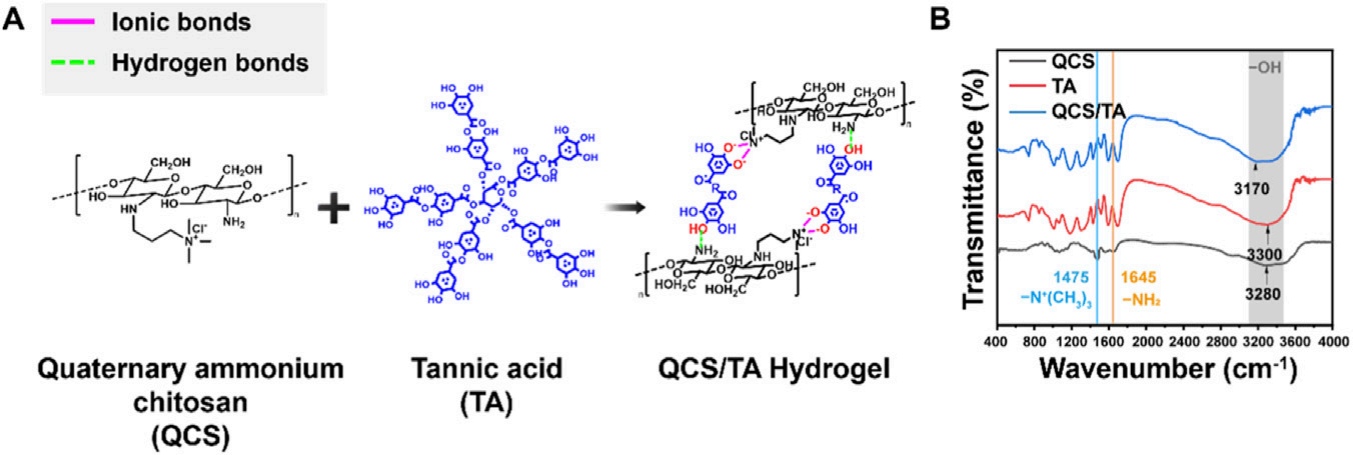
Preparation and characterization of QCS/TA hydrogels. **(A)** Schematic illustration of the crosslinking mechanism. QCS/TA formation was primarily governed by ionic bonds between the quaternary ammonium groups of QCS and the polyphenol groups of TA, as well as hydrogen bonds between the amino groups of QCS and the polyphenol groups of TA. **(B)** FT-IR revealed characteristic peak shifts upon QCS and TA interaction, confirming hydrogel formation via ionic and hydrogen bonding, N = 3, n = 3.

The formation of ionic and hydrogen bonds between the QCS and TA molecules drives the crosslinking and gelation of the QCS/TA hydrogel. Specifically, the trimethyl quaternary ammonium cationic groups (−N^+^(CH_3_)_3_) of QCS serve as positively charged binding sites that can form ionic bonds with the phenoxide anions (−O^−^) generated with ionization of the polyphenolic groups in TA. At the same time, intermolecular hydrogen bonds can form between the amino groups (−NH_2_) of QCS and the polyphenolic groups of TA. With the ionic bonds serving as the primary crosslinking force and the hydrogen bonds as the secondary, those specific interactions together establish a stable 3D network structure of QCS/TA (Figure 1A).

FT-IR was employed to investigate the interactions between QCS and TA. Pure QCS showed characteristic absorption peaks at 1,645 cm^-1^ and 1,475 cm^-1^, which were significantly attenuated or absent in the spectrum of QCS/TA hydrogel. Moreover, the broad absorption bands of pure QCS at 3280 cm^-1^ and pure TA at 3300 cm^-1^ merged and redshifted to 3170 cm^-1^ in the QCS/TA hydrogel (Figure 1B).

**FIGURE 2:**
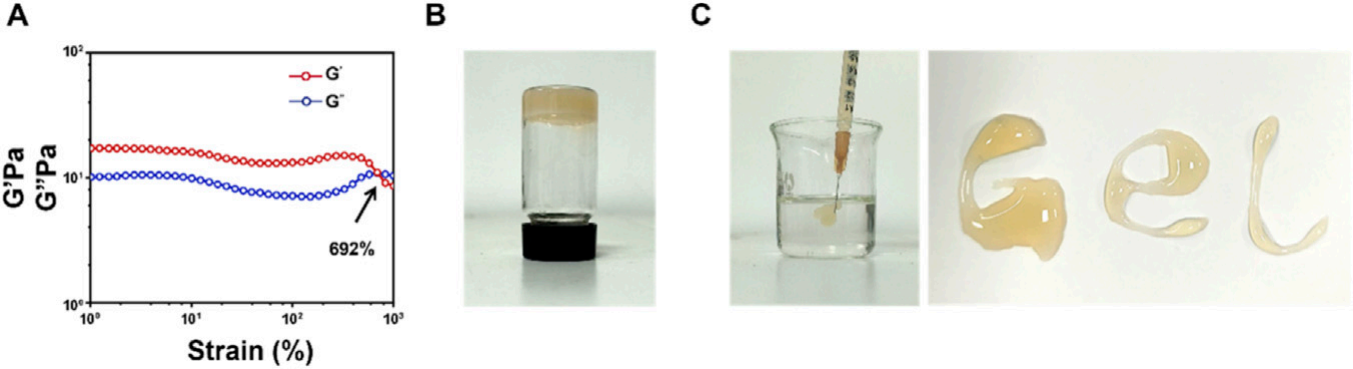
Rheological properties and injectability of QCS/TA hydrogel. **(A)** Strain sweep analysis of the hydrogel (1%–1000% strain), the crossover point occurred at a strain of 692%, N = 3, n = 3. **(B)** Vial inversion method confirming gelation, N = 3, n = 3. **(C)** The hydrogel showed good injectability by smooth extrusion through a 25 G needle, N = 3, n = 3.

Dynamic strain sweep rheological analysis revealed that the storage modulus G′ remained consistently higher than the loss modulus G″ across a strain range of 1%–692% (Figure 2A). Gelation was further confirmed by the vial inversion method, with no observable fluid flow after 30 s (Figure 2B). The hydrogel also demonstrated good injectability, as evidenced by its smooth extrusion from a 25G needle and its ability for pattern writing (Figure 2C).

**FIGURE 3:**
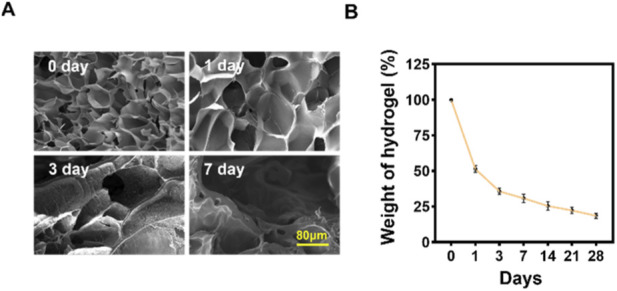
*In vitro* degradation of the QCS/TA hydrogel. **(A)** Representative SEM images of hydrogels after incubation in PBS (pH 7.4, 37 °C) for 0, 1, 3, and 7 days, showing progressive structural change over time, N = 3, n = 3. **(B)** Hydrogel’s *in vitro* degradation profile (pH 7.4 PBS, 37 °C), N = 3, n = 3.

To evaluate QCS/TA’s *in vitro* degradation behavior, the hydrogel samples were lyophilized and weighed after incubation in PBS for 0, 1, 3, 7, 14, 21, and 28 days, and SEM was used to examine the morphology of the gel network. The hydrogel degraded to less than 25% of its original weight within 28 days (Figure 3B). SEM revealed progressive fragmentation of the gel network from day 0 to day 7, accompanied by increased pore size and decreased pore number of the hydrogel (Figure 3A). After day 7, further SEM analysis was precluded due to substantial weight loss of the hydrogel samples causing a significant gel volume reduction and enhanced gel brittleness after lyophilization. SEM results revealed that although the hydrogel network gradually fractured with enlarged pore size and reduced pore number from day 0 to day 7, the material retained a continuous and interconnected porous framework without overall collapse within the first seven days, enabling stable and sustained release of bioactive components from the mADSCs’ lysate during the core therapeutic window. After day 7, continuous degradation of the polymer skeleton rendered the hydrogel brittle and fragile, making it unable to withstand sample preparation for SEM. The significant weight loss of samples at day 28 in the degradation curves further indicated that severe structural damage only emerged after the 7-day lysate release cycle, which would not interfere with the material’s performance during the early myocardial tissue repair phase. The acute and subacute inflammatory injury repair following MI primarily takes place within the first week post-infarction. This hydrogel can continuously release therapeutic lysate-derived vesicles for 168 h while maintaining intact structural support, which highly matches the time window required for early myocardial injury intervention.

### 3.2 Characterization of mADSCs

**FIGURE 4:**
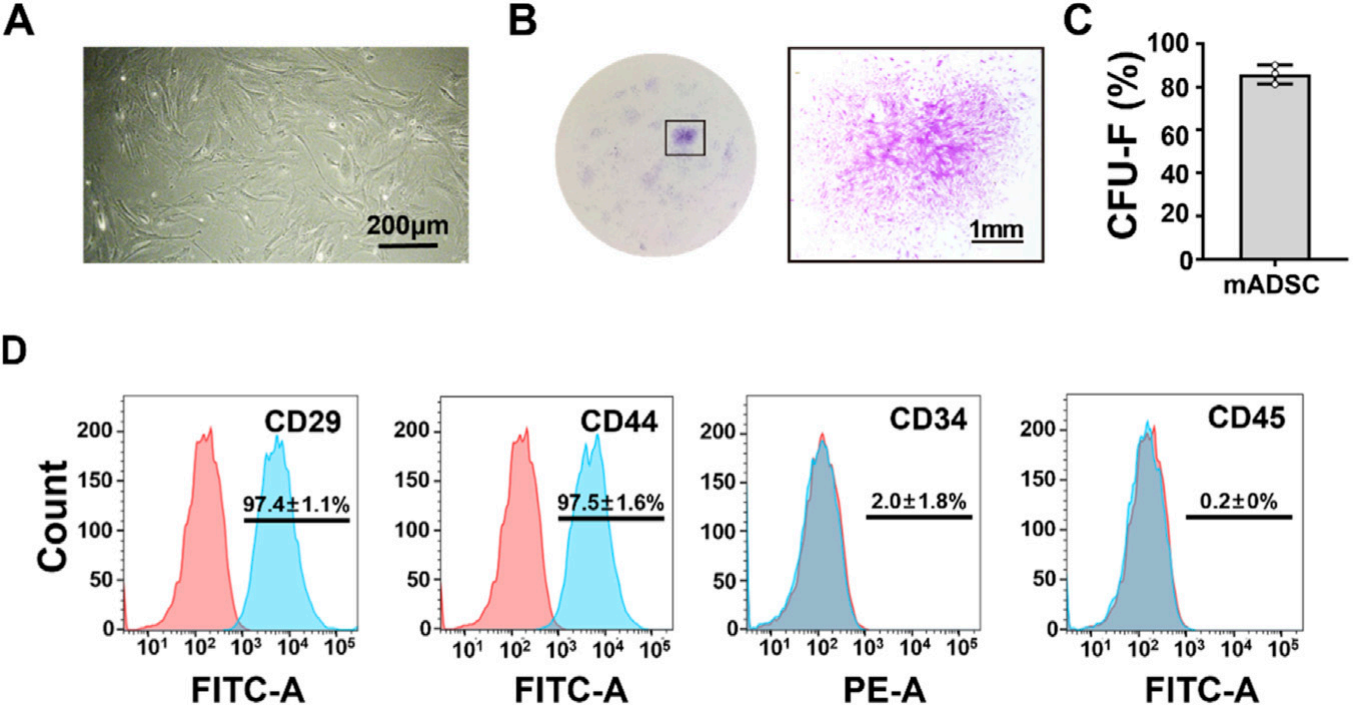
Characterization of mADSCs. **(A)** Morphology of P5 mADSCs, N = 3, n = 3. **(B)** Representative CFU-F colony, N = 2, n = 3. **(C)** CFU-F% of mADSCs. **(D)** Flow cytometry for surface markers (CD29, CD44, CD34, CD45) of mADSCs, N = 3, n = 3.

**FIGURE 5:**
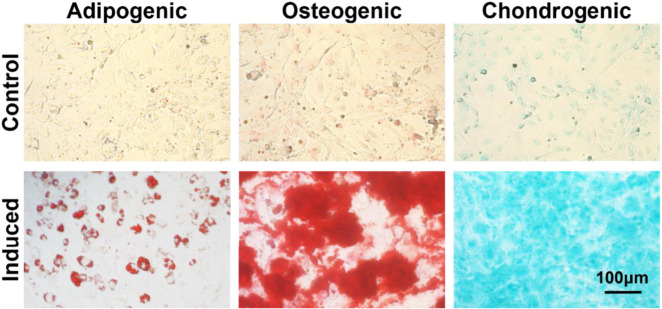
Trilineage differentiation of mADSCs. Representative images of mADSCs control and the ones induced by adipogenic, osteogenic, and chondrogenic differentiation medium. Scale bar = 100 μm. Magnification: 20×, N = 3, n = 3.

mADSCs exhibited adherent growth with a predominantly spindle-shaped morphology (Figure 4A). Colony-forming unit–fibroblast (CFU-F) assay showed a colony formation rate of 85.83% ± 4.39% (Figures 4B,C). Immunophenotype analysis revealed that the mADSCs were positive for CD29 and CD44 (> 90%) and negative for CD34 and CD45 (< 3%) (Figure 4D). Additionally, the mADSCs demonstrated differentiation capacity towards adipogenic, osteogenic and chondrogenic lineages (Figure 5).

### 3.3 Characterization of mADSCs lysate

**FIGURE 6:**
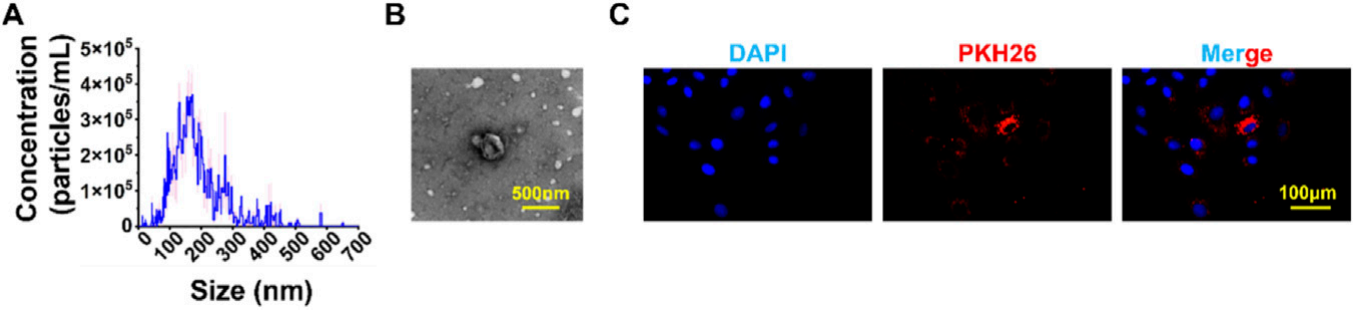
Characterization of mADSCs lysate-derived vesicles and the vesicle uptake by H9c2 cells. **(A)** Size distribution of mADSCs lysate-derived vesicles, N = 3, n = 3. **(B)** TEM image of mADSCs lysate-derived vesicles, scale bar = 500 nm, N = 3, n = 3. **(C)** mADSCs lysate-derived vesicles were labeled with PKH26 fluorescent dye (red fluorescence), and their uptake by H9c2 cells was visualized. The cell nuclei were counterstained with DAPI (blue fluorescence), scale bar = 100 μm, N = 3, n = 4.

NTA was used to evaluate the size distribution and concentration of mADSCs-derived vesicles. The mADSCs-derived vesicles’ diameter ranged from 12.5 nm to 647.5 nm, with a median particle size of 157.7 ± 6.0 nm (Figure 6A). TEM images demonstrated abundant vesicles within the mADSCs lysate (Figure 6B). PKH26 staining further confirmed efficient internalization of mADSCs lysate-derived vesicles by H9c2 cells (Figure 6C).

**FIGURE 7:**
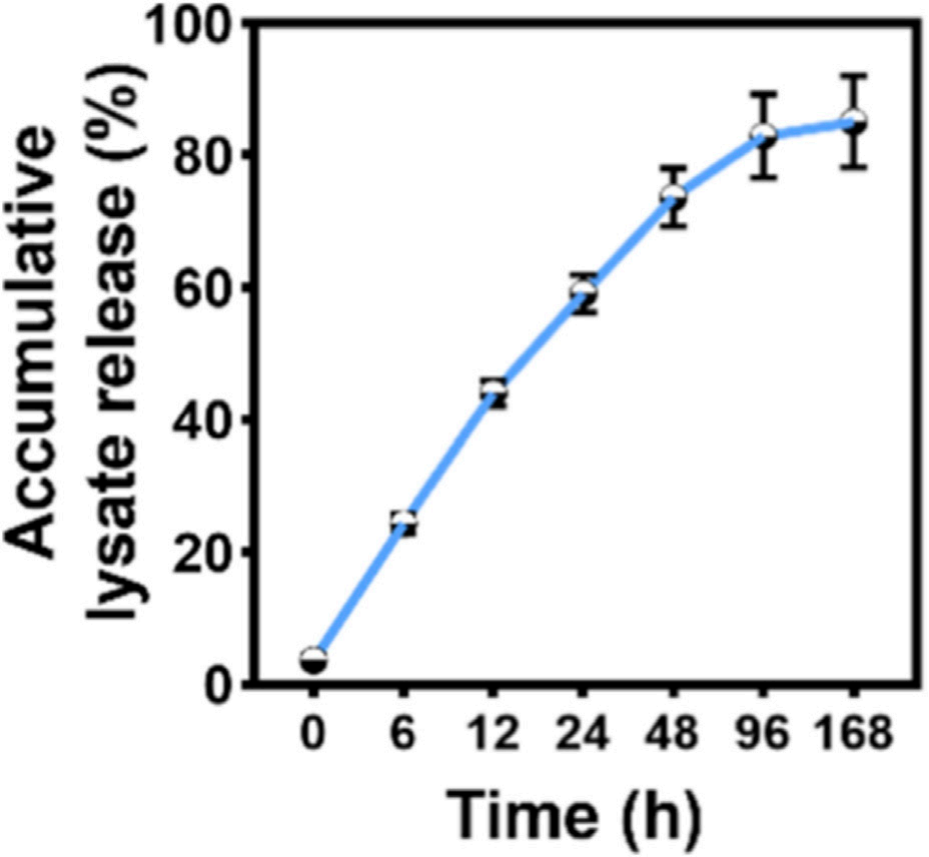
*In vitro* cumulative total protein release profile of mADSCs lysate from the hydrogel (pH 7.4 PBS), showing an initial rapid-release phase followed by sustained release, N = 3, n = 3.

The mADSCs lysate-derived vesicles were encapsulated in the QCS/TA hydrogel, and cumulative protein release was measured at 0 h, 6 h, 12 h, 24 h, 48 h, 96 h, and 168 h time points. The results showed that the protein release increased over time, reaching 85% of the total encapsulated protein release by 168 h (Figure 7). Meanwhile, the QCS/TA hydrogel exhibited a biphasic release profile, with an initial burst release followed by sustained delivery for up to 168 h. Immune activation, endothelial dysfunction, and cardiomyocyte injury arise in the early phase after myocardial infarction, and these can be alleviated by the high concentrations of bioactive factors released during the initial burst phase. The subsequent long-term sustained release continuously modulates angiogenesis, extracellular matrix remodeling, and inflammatory responses, matching the dynamic pathophysiological process of myocardial repair.

**FIGURE 8:**
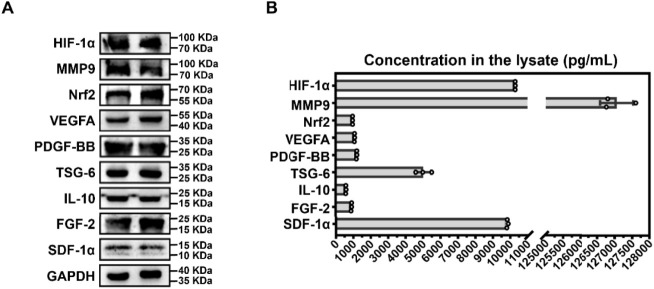
Cytokines/growth factors expression profile of mADSCs lysate. **(A)** Western blot analysis of cytokines/growth factors in the mADSCs lysate, N = 3, n = 2. **(B)** Concentrations of cytokines/growth factors in the mADSCs lysate, N = 3, n = 2.

Western blot and ELISA results showed that the mADSCs lysate contained a panel of growth factors/cytokines associated with myocardial repair, including HIF-1α, MMP9, Nrf2, VEGFA, PDGF-BB, TSG-6, IL-10, FGF-2, and SDF-1α (Figures 8A,B). Specifically, HIF-1α, MMP9, and SDF-1α were present at high concentrations in the mADSCs lysate (Figure 8B). Those growth factors/cytokines are known to mediate key processes in cardiac repair and regeneration, such as angiogenesis, immunomodulation, anti-oxidation, and anti-apoptosis (Broughton et al., 2018). Collectively, those findings indicated that mADSCs lysate is enriched with diverse growth factors/cytokines, which can be efficiently internalized by target cells, supporting its potential as a cell-free therapeutic strategy for treating myocardial tissue damage.

The vesicles derived from mADSC lysates cannot be classified as typical extracellular vesicles (EVs). According to the general EV research guidelines, EVs are lipid-bilayer particles that are naturally secreted by cells into the extracellular space (Théry et al., 2018). The vesicles derived of this study were obtained from the lysed mADSCs and therefore should belong to IVs. Based on the method we used for IVs isolation (100,000 g, 70 min), we aimed to retain the IVs as much as possible while removing large cell membrane fragments and the protein aggregates. However, a tiny amount of smaller cell membrane fragments and protein aggregates could still inevitably remain. In addition, whole lysates were used in the experiments as it was believed that the therapeutic effect can be mediated by the synergistic action of multiple components that include mADSCs-derived IVs and various cytokines. Our isolation method was based on the study by Xingxiang Duan et al. (Duan et al., 2024), who found that vesicles derived from the dental pulp MSCs’ lysates exhibited biological characteristics similar to those of EVs. Specifically, dental pulp MSCs-derived vesicles also expressed EV-specific markers, including Alix, TSG101, CD9 (Duan et al., 2024). Similar to this previous study, our characterization data also showed that the mADSCs lysate-derived vesicles of this study also expressed those EV-specific markers (TSG101, CD81 and CD9) (Supplementary Figure 1).

### 3.4 The effects of QCS/TA hydrogel and mADSCs lysate on the metabolic activity and cell number of H9c2 cells, L929 cells, and HUVECs

**FIGURE 9:**
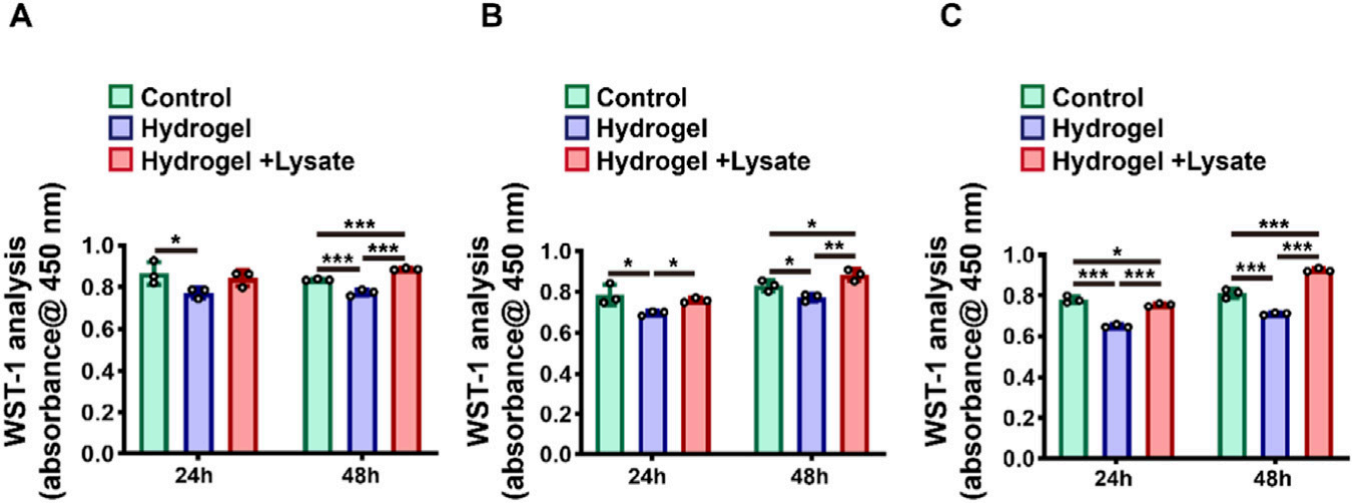
Effects of hydrogel and hydrogel carrying mADSCs lysate on the metabolic activity of **(A)** H9c2 cardiomyocytes, **(B)** L929 fibroblasts and **(C)** HUVECs. *p < 0.05, **p < 0.01, ***p < 0.001, (N = 3, n = 2).

**FIGURE 10:**
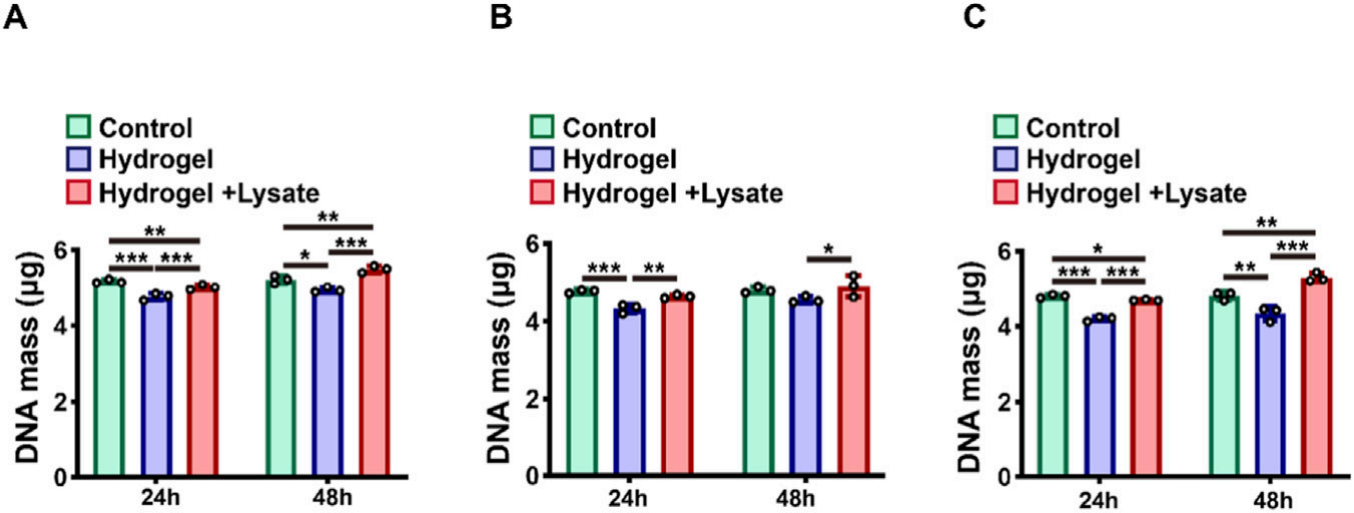
Effects of hydrogel and hydrogel carrying mADSCs lysate on the cell number of **(A)** H9c2 cardiomyocytes, **(B)** L929 fibroblasts, and **(C)** HUVECs. *p < 0.05, **p < 0.01, ***p < 0.001, (N = 3, n = 2).

Finally, the feasibility of using the QCS/TA hydrogel as a delivery vehicle for mADSCs lysate in the context of cardiac repair was evaluated. Specifically, three cell types including H9c2 (cardiomyocytes), L929 (fibroblasts), and HUVECs (endothelial cells) were treated with hydrogel alone or hydrogel carrying mADSCs lysate for 24 h and 48 h. The effects of the hydrogel and hydrogel carrying mADSCs lysate on the metabolic activity and cell number of those three types of cells were investigated (Figures 9, 10).

WST-1 results showed that H9c2 cells, L929 fibroblasts and HUVECs’ metabolic activity decreased with the hydrogel treatment relative to the control group (Figure 9). Nevertheless, metabolic activity remained above 70% of the control level in all hydrogel-treated groups, exceeding the cytotoxicity threshold defined in ISO 10993, which supports acceptable *in vitro* cytocompatibility. Further, the addition of mADSCs lysate alleviated the mild reduction in metabolic activity induced by the hydrogel alone, indicating the cytoprotective effect of the mADSCs lysate (Figure 9) across all three cell types.

DNA quantification further revealed that the hydrogel alone slightly reduced cell number across the three cell types, whereas the hydrogel carrying mADSCs lysate effectively attenuated this small negative effect (Figure 10). Collectively, findings of the cell metabolic activity assay and the cell number quantification assay demonstrated that the hydrogel held promise as a feasible delivery platform for mADSCs lysate in cardiac tissue repair (Figures 9, 10).

## 4 Discussion

MI, which is defined as the most common manifestation of coronary heart disease, remains a leading cause of death worldwide (Anderson and Morrow, 2017). MI is typically initiated by coronary thrombosis, which then impairs myocardial perfusion and triggers prolonged ischemia/hypoxia, ultimately leading to extensive cardiomyocyte necrosis (Reed et al., 2017). Although reperfusion is the standard clinical intervention post-MI, it paradoxically induces myocardial ischemia-reperfusion injury (MIRI) (Bahit et al., 2018). The pathogenesis of MIRI often involves oxidative stress, calcium dysregulation and inflammation, which could further cause fibrosis and abnormal ventricular remodeling (Anderson and Morrow, 2017; Reed et al., 2017). Those interconnected pathological processes emphasize the need for therapeutic strategies capable of simultaneously addressing multiple facets of MIRI.

ADSCs have gained great interest for cardiovascular tissue repair due to their ease of isolation, high expansion ability and robust paracrine signaling activity (Guan et al., 2025). ADSCs could secrete pro-angiogenic and anti-inflammatory factors that recruit endogenous endothelial cells and support post-MI tissue healing. In addition, ADSCs also have the potential to differentiate towards cardiomyocyte-like cells (Roura et al., 2017). In the current study, mADSCs were isolated from the murine inguinal adipose tissue and they exhibited typical spindle-shaped morphology. The mADSCs showed robust colony-forming ability, high expression of mesenchymal markers (CD29, CD44), low expression of hematopoietic markers (CD34, CD45), and demonstrated adipogenic, osteogenic and chondrogenic differentiation capacity, confirming their MSC identity.

Despite the promise of MSC-based therapies, their clinical translation is hindered by several limitations including poor survival in the local tissue microenvironment, potential tumorigenicity and risks of embolism (Roura et al., 2017). Recent studies have indicated that the therapeutic benefits of MSCs are mainly derived from their paracrine signaling activities, mediated by the growth factors, cytokines and microRNAs that the MSCs secrete (Qin et al., 2023). On the other hand, MSCs’ lysate could contain not only the cell secretome but also the large amount of intracellular growth factors, cytokines and miRNAs that are synthesized but not secreted by the MSCs (Zununi Vahed et al., 2025). Notably, the yield of intracellular vesicles (IVs) from cell lysate can be ten times higher than that of extracellular vesicles (EVs) from cell conditioned medium, and IVs carry a significantly higher protein load vs. EVs (Zununi Vahed et al., 2025). In the current study, the mADSCs lysate yielded 1.2 × 10^13^ particles/mL and PKH26 labeling showed efficient internalization of mADSCs lysate-derived vesicles by H9c2 cells, confirming their bioactivity. Western blot and ELISA further identified a broad spectrum of functional cytokines/growth factors in the mADSCs lysate, including pro-angiogenic (SDF-1α, MMP9, PDGF-BB, VEGFA, FGF-2), anti-inflammatory (IL-10, TSG-6), anti-fibrotic (FGF-2) and antioxidant (TSG-6, HIF-1α, Nrf2) factors/cytokines. Those findings suggested that mADSCs lysate has great potential to promote angiogenesis, suppress inflammation and fibrosis, and mitigate oxidative stress concurrently, which are all key processes for myocardial tissue repair and regeneration. Among the identified factors, VEGFA, FGF-2, PDGF-BB, MMP9 and SDF-1α may primarily contribute to endothelial cell support and angiogenic responses (Seghezzi et al., 1998; Petit et al., 2007; Mahecha and Wang, 2017; Hosaka et al., 2018), whereas IL-10 and TSG-6 may be more involved in immunomodulation and the regulation of fibroblast behavior (Ouyang et al., 2011; Albtoush et al., 2023). Activation of HIF-1α and Nrf2-related antioxidant signaling may also help explain the partial preservation of metabolic activity in H9c2 cardiomyocyte-like cells under hydrogel/lysate treatment (Ngo and Duennwald, 2022; Zhang et al., 2025). These mechanistic associations remain hypotheses and warrant direct investigations in future studies.

To enable localized and sustained delivery of the mADSCs lysate, an injectable QCS/TA hydrogel was developed according to previously established protocol (Guo et al., 2022b; 2022a). Chitosan, which is a naturally-occurring polysaccharide that shares similar structure to glycosaminoglycans, is suitable for myocardial tissue engineering (Kim et al., 2023). Quaternization can further increase the charge density and hydrophilicity of chitosan while preserving its inherent biocompatibility and biodegradability (Kim et al., 2023). On the other hand, TA is a polyphenol derived from plants and it can serve as a dynamic crosslinker enabling abundant ionic and hydrogen bonds formation with QCS (Moghaddam et al., 2022). Specifically, the ionic bonds formed between the QCS’s quaternary ammonium groups and TA’s phenoxide anions, together with hydrogen bonds formed between the QCS’s amino groups and TA’s phenolic groups, generate a stable 3D hydrogel network. FT-IR analysis of the study confirmed those interactions, as evidenced by the attenuation of characteristic peaks for amino and quaternary ammonium groups and the redshift of -OH absorption bands. Rheological analysis further validated the hydrogel’s network stability, with the storage modulus G′ exceeding the loss modulus G″ across a strain range of 1%–692%, indicating the formation of an elastic gel. The hydrogel could be squeezed out through a 25G needle, demonstrating good injectability of the hydrogel and its potential suitability for intramyocardial delivery.

An ideal delivery vehicle for myocardial tissue repair should exhibit good biocompatibility, biodegradability, as well as support sustained release (Li et al., 2022). This study showed that the hydrogel degraded to less than 25% of its original weight within 28 days *in vitro*, with progressive 3D network fragmentation and hydrogel pore enlargement. This degradation profile aligned well with the time frame required for early-stage myocardial tissue repair and supported sustained release of the encapsulated mADSCs lysate. The degradation products of QCS/TA hydrogels could include soluble QCS fragments, TA-associated phenolic species, and QCS/TA complexes. Although the present study focused on dry-weight degradation and did not chemically identify these products, their concentration-dependent bioactivity and possible effects on cell adhesion, protein adsorption from the medium, osmotic balance, and inflammatory responses should be thoroughly evaluated in future work. Moreover, the study demonstrated that exposure to the hydrogel did not compromise the metabolic activity or proliferation of H9c2 cardiomyocytes, L929 fibroblasts, or HUVECs, with metabolic activities consistently exceeding 70%, meeting the ISO 10993 standard for biomedical materials. Moreover, it was found that the hydrogel carrying mADSCs lysate could effectively enhance the metabolic activities and proliferation of all three types of cells, indicating that the hydrogel has potential as a delivery platform for mADSCs lysate in cardiac tissue repair.

However, the hydrogel alone consistently reduced metabolic activity and DNA content across all tested cell types, which could be attributed to multiple factors including TA concentration, released components of the hydrogel, osmotic effects of the hydrogel extract, mass transfer limitations, altered cell adhesion behaviors and the hydrogel degradation products. Firstly, the biological effects of TA (such as antioxidant, anti-inflammatory and pro-repair effects) are concentration-dependent (Ninan et al., 2016; Chen et al., 2019; He et al., 2020). Higher concentrations of TA or its continuous exposure can reduce cell viability by forming complexes with serum proteins, creating oxidative stress and DNA damage (Wang et al., 2019). Thus, a small amount of free TA release or dynamically dissociated TA during the hydrogel extraction may partially account for a mild decrease in cell metabolic activity and proliferation. Additionally, the cationic QCS could interact favorably with negatively charged cell membranes and medium proteins. High concentrations of QCS derivatives or a high degree of quaternization could reduce cell viability (Haas et al., 2005; Opanasopit et al., 2008). Therefore, if soluble QCS fragments or QCS/TA complexes are present in the hydrogel extract, they may mildly affect cellular metabolism and cell proliferation. Moreover, charged components and polyphenol-polymer complexes in the hydrogel extract could alter the osmotic pressure or ionic strength of the culture medium. Cells are sensitive to osmotic pressure changes and a hypertonic environment could induce cell shrinkage, decreased metabolic activity, cell cycle arrest and apoptosis (Wang et al., 2011). Although the hydrogel extract was filtered through a 0.22 μm membrane, filterable QCS/TA fragments, small released molecules, or nano-sized complexes may still influence medium viscosity, protein adsorption and nutrient diffusion, thus interfering with cell metabolic activities and proliferation (Xie et al., 2017). Furthermore, TA can form complexes with serum proteins (Chen et al., 2022), while QCS can interact with cell membrane or adhesion proteins through electrostatic interactions (Aibani et al., 2021). As a result, the soluble components in the hydrogel extract may cause a slight reduction in cell adhesion and spreading by altering the function of adhesion proteins or via weak interactions. Finally, TA can be hydrolyzed to gallic acid (Curiel et al., 2010), which exhibits concentration-dependent cytotoxicity, causing ROS production, cell cycle arrest and apoptosis (Choubey et al., 2018). All those factors may together contribute to the mild inhibitory effect of the hydrogel on cell metabolic activity and DNA content.

Overall, this is the first study that explored combining an injectable hydrogel with ADSCs lysate for potential myocardial tissue repair. This cell-free platform could offer several advantages such as circumventing the safety issues associated with live MSCs transplantation, providing a rich and versatile cocktail of bioactive growth factors and cytokines and enabling a minimally invasive, localized and sustained delivery approach. However, some considerations warrant further investigations of the current study. Specifically, due to the complexity of the content of the ADSCs lysate, careful optimization should be performed to define the optimal dosing regimen and release kinetics of the ADSCs lysate. Another limitation of the study is that the release profile was measured using total protein concentration only, this did not demonstrate that biologically active vesicles or cytokines remained functionally intact after release. Subsequent experiments including vesicle-specific particle tracking, enzyme-linked immunosorbent assay of cytokines released from the hydrogel and functional validation tests of the vesicles/cytokines need to be performed in future studies. Additionally, while the *in vitro* results are encouraging, further validations in MI small-animal models would be valuable to assess the *in vivo* safety, translational potential, as well as some of the underlying mechanisms of this cell-free platform. Further, the long-term biocompatibility and biodegradation behavior of the hydrogel within the native cardiac microenvironment should also be studied. Beyond those aspects, the scalability of the hydrogel + ADSCs lysate system and its applicability within patient-specific or allogeneic settings are promising directions that warrant continued research. Altogether, the present study has established a critical basis upon which future translational efforts can be built. For future clinical translation, key considerations include defining a safe and effective intramyocardial injection volume, comparing intramyocardial, epicardial, and intracoronary delivery routes, translating the *in vitro* effective concentration into an appropriate *in vivo* dose based on cardiac tissue volumes in animals and humans, and determining whether autologous or allogeneic ADSC lysate provides the most practical balance between personalization, manufacturing consistency, and off-the-shelf availability.

## 5 Conclusion

In conclusion, this study demonstrated that ADSCs lysate encapsulated in an injectable hydrogel could be a promising cell-free therapeutic strategy for myocardial tissue repair. This cell-free platform combined the broad regenerative potential of ADSCs lysate with the biocompatible and biodegradable hydrogel, offering a promising delivery approach that could address multiple pathological issues post-MI. The findings of the study provide a critical *in vitro* foundation for further *in vivo* investigations for post-MI treatment.

## Impact statement

This study presents a cell-free hydrogel-based platform delivering adipose-derived mesenchymal stem cell lysate-derived vesicles for potential myocardial tissue repair. The platform leverages the rich anti-inflammatory, pro-angiogenic and antioxidant cytokines/growth factors repertoire of adipose-derived mesenchymal stem cell lysate, delivered locally via an injectable hydrogel with excellent biodegradability and biocompatibility. This method circumvents the safety issues associated with live cell transplantation for cardiac repair and could offer a scalable, cell-free delivery strategy for myocardial repair-related applications, establishing a foundation for future *in vivo* investigations and further translational evaluation.

## Data Availability

The raw data supporting the conclusions of this article will be made available by the authors, without undue reservation.
