# Supplementary material for: A hydrogel platform delivering mesenchymal stem cell lysate for potential myocardial tissue repair
Source: Front Bioeng Biotechnol. 2026 Jul 31;14:1855161. doi: 10.3389/fbioe.2026.1855161 (PMC13473002; doi:10.3389/fbioe.2026.1855161)
Supplement: Supplementary file 1 [file Supplementaryfile1.docx]

**Supplemental Figure 1**. WB characterization of mADSCs lysate-derived vesicles. GAPDH serves as an internal control, N = 3, n = 3.
